# Metasurface Loaded High Gain Antenna based Microwave Imaging using Iteratively Corrected Delay Multiply and Sum Algorithm

**DOI:** 10.1038/s41598-019-53857-0

**Published:** 2019-11-21

**Authors:** M. Tarikul Islam, Md. Samsuzzaman, Salehin Kibria, Norbahiah Misran, Mohammad Tariqul Islam

**Affiliations:** Department of Electrical Electronic and Systems Engineering, Faculty of Engineering and Built Environment, Universiti Kebangsaan Malaysia, Bangi, 43600 Selangor Malaysia

**Keywords:** Biomedical engineering, Electrical and electronic engineering, Imaging techniques

## Abstract

In this paper, the design consideration is investigated for a cylindrical system with low-cost and low-loss dielectric materials for the detection of breast tumor using iteratively corrected delay multiply and sum (IC- DMAS) algorithm. Anomaly in breast tissue is one of the most crucial health issues for women all over the world today. Emergency medical imaging diagnosis can be harmlessly managed by microwave-based analysis technology. Microwave Imaging (MI) has been proved to be a reliable health monitoring approach that can play a fundamental role in diagnosing anomaly in breast tissue. An array of 16 high gain microstrip antennas loaded by Index Near-Zero (INZ) metasurfaces (MS), having the impedance bandwidth of 8.5 GHz (2.70–11.20 GHz) are used as transceivers for the system. The MS is used to increase the electrical length of the signal that results in the gain enhancements. The antennas are mounted in a cylindrical arrangement on a mechanical rotating table along with a phantom mounting podium. A non-reflective positive control switching matrix is used for transmitting and receiving microwave signals. A set of lab-made realistic heterogeneous breast phantoms containing skin, fat, glandular, and tumor tissue dielectric properties in individual layers are used to verify the performance of the proposed technique. The control of the mechanical unit, data collection, and post-processing is conducted via MATLAB. The system can detect multiple tumor objects. The imaging results and numerical Signal to Mean Ratio (SMR) values of the experiment validate the system efficiency and performance that can be a viable solution for tumor detections.

## Introduction

**Figure 1:**
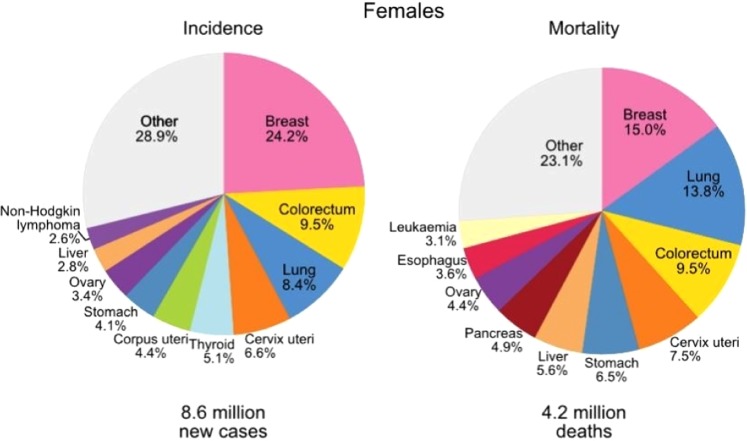
Pie Chart of recorded 2018 woman cancer cases^7^.

Breast cancer is believed as one of the significant health issues across the world that develops from breast tissue. About 25% of cases of diseases among women are the causes of breast cancer globally each year^1,2^. Above 2 million breast cancer cases are diagnosed worldwide every year, and the death rate is alarming. The survival rate can be drastically increased by early detection that allows urgent treatment for recovery as well as a comfortable diagnosis. In 2018, GLOBALCAN recorded 8.6 million cancer cases for women, among them, maximum cancer cases (24.2%) were identified from breast cancer, as shown in Fig. 1.

X-ray mammography, ultrasound imaging (USI), magnetic resonance imaging (MRI) are the conventional methods for the detection of breast cancer. But the missed detection rate is quite high in some cases up to 4% to 34%^3^. Likewise, ionization from the X-ray mammography has a severe threat to health; even repeated examination could develop cancer inside women’s body^4^. In most cases, patients feel uncomfortable for unusual breast compression during the diagnosis process. A non-ionizing and more sensitive way of detection is introduced as an MRI for detecting breast cancer with high efficiency, but the main drawbacks are expensiveness and low specificity, which cause inaccurate diagnoses^5^. Another alternative method is USI that has 17% of false detection record^6^. Consequently, an easy, inexpensive, and non-ionizing method is highly desirable for the systematic assessment of breast health.

Recent studies show that the electrical properties of normal and malignant cells have a significant difference^3,8^. This key factor is exploited in microwave imaging (MI) techniques in breast health monitoring for its wide range of advantages^9–11^. Because of having no ionizing radiation, it can be a promising candidate as a replacement for X-ray mammography. Primarily two significant types of imaging are used for various purposes, one is microwave tomography^12^, which maps the distribution of dielectric properties of the object; and another one is radar-based imaging^13,14^ that maps the position of brightest scattering objects in the imaging domain. The antenna acts as the transducer in the MI system where one antenna transmits a low power microwave signal through the target, and the other antennas receive the scattered signals. The scattered signals vary due to the different dielectric properties of healthy and cancerous tissue. Microwave imaging draws attention to this field as it can detect the tumors with different sizes and locations with an accuracy of up to 90%^15^.

Microwave imaging works on two principal methods, radar-based techniques, and microwave tomography. The radar-based technologies work on mapping the breast tissue by analyzing backscattering signals affected by a different dielectric of tissues. In microwave tomography, nonlinear and ill-posed contrary scattering signals are calculated to reconstruct the breast properties. The practical measurements can be inspected in both the frequency domain and the time domain^16^. Among them, the frequency domain method is better because the inadequate signal to noise ratio is displayed in the time domain. Currently, two types of systems used for breast cancer detection, which are multistatic and monostatic radar-based systems, are being investigated widely. The multistatic system consists of an antenna array and a complex switching network^17^. Most of the cases, more than four antenna units are used to collect scattered signals to form a high-resolution image. Additionally, the accuracy is enhanced by using lower frequency signals that can penetrate deep inside the human tissue, but this results in lower resolution image. To overcome this problem, the multi-static radar-based system helps to improve the resolution with a complex switching network, which captures more data and additional features. On the other hand, the monostatic system uses a single or a pair of antennas and works on a higher frequency above 4 GHz. Therefore, the antenna performance relates to bandwidth, gain, radiation pattern directionality, and antenna efficiency.

In recent years, researchers reported several body area networks^18,19^ and broadband antenna based health monitoring system, including breast tumor detection^10,17,20–22^. Up-To-Date technology is the reflection of the recent comprehensive imaging system for medical applications. A tomography system consisting of an array of 16 monopole antenna were developed by Meaney *et al*. in 2014^23^, where the authors propose an updated rotating bi-static angle-bent antenna^24^. In the system, the patient lies in the horizontal posture, and the antenna array is set to vertical orientation surrounding the breast to collect the monotonous microwave signals. A forward solver then calculates the simulated signals and compares to the measured data to estimate the property of the breast. A multi-static radar-based imaging system was proposed by Klemm *et al*. where the UWB antenna array was used for sensing^25,26^. The antennas are positioned in a semicircular layout, and while running the experiment, an integrated short pulse is generated with a vector network analyzer. The Delay and Sum (DAS) algorithm is used to reconstruct the breast image to identify the tumor from the reflected signals. Preece *et al*. reported the breast tumor detection prototype for clinical evaluation^27^. A mono-static radar-based imaging system has been proposed by Fear *et al*. using a single Vivaldi antenna that is attached to a mechanical arm^28,29^. By using the DAS algorithm, the image is generated from the signals recorded from multiple antenna positions like the multi-static method. Several clinical trials have been run using the system and the results were presented in literature^30^. Furthermore, Porter *et al*. have developed a time-domain system consisting of an antenna array for breast examination^31^. The received signals are analyzed, and the relative permittivity of the breast tissue is estimated. By determining the variation in dielectric properties, the presence of malignant cells is predicted.

Different kinds of methods like metamaterial structures^32,33,46,47^, metalens^34^, electromagnetic bandgap structure (EBG)^35^, artificial magnetic conductor (AMC)^36^, metadeflectors^37^, and metasurfaces^38,39^ have been introduced to modify the Electromagnetic wave for performance improvement. For the enhancement of the bandwidth, gain and isolation capabilities of antenna parameter, metamaterial structure, and split ring resonators are drawing attention day by day. Metamaterial denotes an artificial material construction with a special kind of electromagnetic properties that do not exist in nature. A metamaterial structure can be fitted in the antenna ground for increasing isolation between the antenna radiators that results in either negative permeability, permittivity or both (double negative metamaterial) at the same time. It can control the electromagnetic waves and efficiently transmits the signals due to its negative properties. Several additional designs with different behaviors have been proposed earlier, including near-zero permittivity or Epsilon-Near-Zero (ENZ)^32^. Near zero permeability is familiar as mu-near zero (MNZ), which is a special property of metamaterial that draws much interest in microwave applications^33^. This structure has the frequency-dispersive efficient permeability that can turn out to be zero at a specific frequency. This properties of MNZ makes the structure well suited for several attractive microwave application, including metamaterial lens and absorbers. Several left-handed metamaterials were reported having different types of structure like SRRs^40^, Multiple SRRs^41^, fishnet structures^42^, spiral SRRs^43^, double-sided SRRs^44^, cut wire pairs^45^, broadside-coupled SRRs^46^, etc. The substantial broad bandwidth is achieved by using non-resonant metamaterial structures^47,48^. By implementing metasurfaces the antenna gain has improved^38^. The MS was used to manipulate the main and side lobes of the Vivaldi antenna. An alternative for a beamforming array antenna is proposed by introducing MS that is simpler and economical^39^. The design of arbitrary bifunctional CP meta-devices was introduced by implementing unit cell of four-layer metallic patches^49^. To achieve the narrow frequency band, the unit cell spectrum and the range are being curbed and make the complexity of fabricating and implementing in antennas. However, these problems have been investigated and addressed with varying degrees of success, resulting in expansion of the metamaterial utilization on various aspects electromagnetic applications including antenna performance enhancement.

In this paper, the authors presented an imaging system prototype for monitoring breast health and early detection of breast tumors. The system employs a 16-antenna array, where eight antennas act as transmitters and another eight antennas act as receivers. The antenna is inspired by index near zero metasurfaces, which helps to achieve high gain and directional radiation. The proposed IC-DMAS algorithm is used to generate the high-resolution images from the lab-made heterogeneous breast phantom screening. The images with identified multiple tumor objects are also presented. The numerical SMR values are also presented to validate system performance.

## Imaging System Setup

### System design

**Figure 2:**
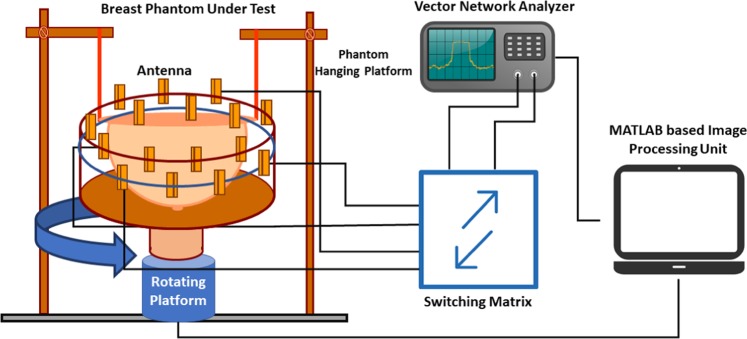
Imaging system block diagram.

The imaging system aims to recognize the variations in backscattered signal with the lab-made heterogeneous phantom that holds different dielectric properties of human breast tissue (skin, fat, gland), including the tumor with high dielectric. A computerized system is developed where a single computer controls all the units where antenna work as a transceiver and the scattered signals are accumulated by a Vector Network Analyzer (VNA). The block diagram of the proposed imaging system is shown in Fig. 2. The MI system comprises of a 16-metasurface loaded antenna array, two switching matrices (9-port each), a mechanical rotating platform, VNA, the MATLAB based signal processing as well as image reconstruction algorithm. The antennas are installed in a cylindrical arrangement where the transmitting and receiving antennas are interleaved. The lab-based breast phantom is placed inside the array. The turntable rotates the array in a complete rotation around the phantom for taking every 7.2-degree scanned position of in total (8 × 8 × 50) data. N*φ* = 50 equally spaced points then collect the data. The complex S-parameters (frequency domain), S (*f, tx, rx, φ*) data is captured, where *f* denotes the frequency, *rx* = 1, 2… 8 and *tx* = 1, 2… 8 are the receiving and transmitting antennas, respectively, and *φ* is the orientation of the rotating platform. The system operates in the 2.7 to 8.0 GHz bandwidth with 201 points evenly spaced through the band.

### Antenna design

#### Metasurface unit cell design

**Figure 3:**
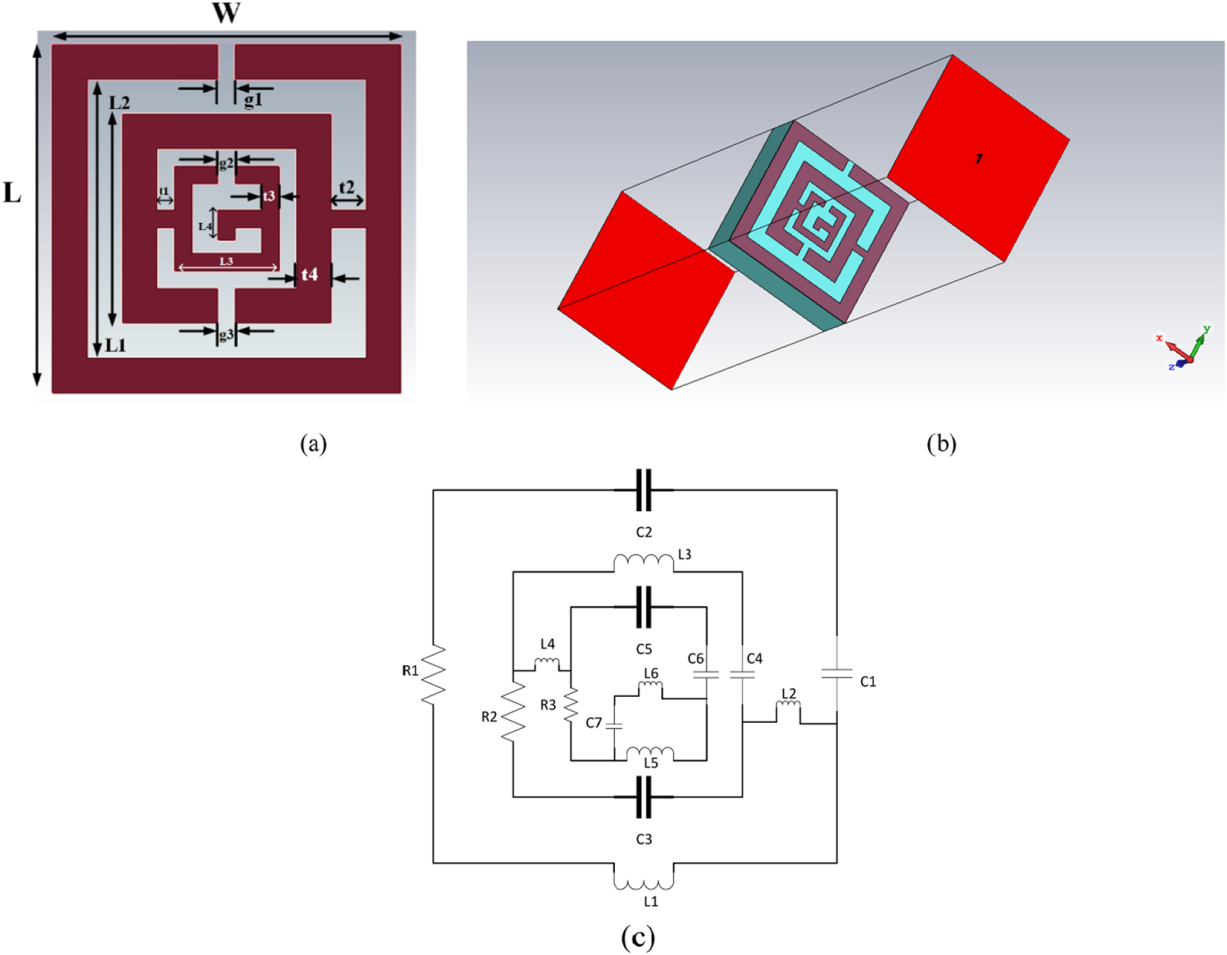
(**a**) Top view of MS unit cell, (**b**) Simulation setup, (**c**) Equivalent circuit diagram. (Optimized parameters: *W* = *5 mm, L* = *5 mm, L1* = *4 mm, L2* = *3 mm, L3* = *1.5 mm, L4* = *0.43 mm, t1* = *0.25 mm, t2* = *0.5 mm, t3* = *0.25 mm, t4* = *0.5 mm, g1* = *g2* = *g3* = *0.25 mm*).

The antenna design starts with designing a metasurface (MS) unit cell. The main target is to design a unit cell allowing resonance in the ultra-wideband (UWB) frequency range (3.1–10.6 GHz). The miniature structure is achieved by employing a complementary split-ring resonator (CSRR). The split-ring resonator (SRR) is conventional in metasurface that is artificially produced to generate anticipated magnetic predisposition in numerous kinds of MS up to 200 THz^50^. Two opposite concentric split rings construct the SRR structure that is magnetically resonant and induces a vertical magnetic field. This vertical magnetic field often infers negative permittivity values. The split gap between the ring produces capacitance that helps to control the resonance of the structure. The CSRR proposed in this paper is the combination of multiple SRR structures, which gives more control over resonance and is illustrated in Fig. 3(a). The simulation is conducted using Computer Simulation Technology (CST) software in the finite-difference time-domain (FDTD) method to analyze S-parameters. Rogers RT5880 is used as a substrate which has a permittivity, loss tangent, and thickness of 2.2, 0.0009 and 1.57 mm, respectively. Figure 3b shows orientation of the simulation setup. The equivalent circuit diagram of the unit cell is illustrated in Fig. 3(c). This unit cell has been developed in terms of the transmission line principle, where a single patch act as a series RLC circuit. This type of structure is analyzed and utilized as a passive LC circuit that interacts with a single resonant frequency, which is governed by the following equation,

1 $$f=\frac{1}{\pi \sqrt{{\rm{LC}}}}$$

where *L* denotes the lumped inductance, and *C* denotes the lumped capacitance. The split inside the metal loop represents capacitance, and the metal loops form inductance.

The combination of the split and electric field generates electric resonance; on the other hand, the magnetic fields and the metal loops form magnetic resonances when electromagnetic wave illuminates the structure. The formation of capacitance between the split can be explained with the following equation,

2 $$C={\varepsilon }_{0}{\varepsilon }_{r}\frac{A}{d}(F)$$

here, *ε*_0_ represents free space, and *ε*_*r*_ is relative permittivity, the area of the split is *A*, and *d* stands for the split length which is “*g*” in the proposed structure. The equivalent inductance is calculated according to the transmission line principle mentioned in literature^51^.

3 $$L(nH)=2\times {10}^{-4}l\,[\mathrm{ln}(\frac{l}{w+t})+1.193+0.02235\frac{w+t}{l}]\,{K}_{g}$$

where, *l* = length of microstrip line, *w* = width of microstrip line, *t* = thickness of microstrip line, correction factor = *Kg* = 0.57–0.145 ln ${\rm{In}}\frac{w^{\prime} }{h^{\prime} },w^{\prime}$ means the thickness of the substrate and *h*′ means the width of the substrate. Both the external and internal inductance must be considered to determine the total inductance.

**Figure 4:**
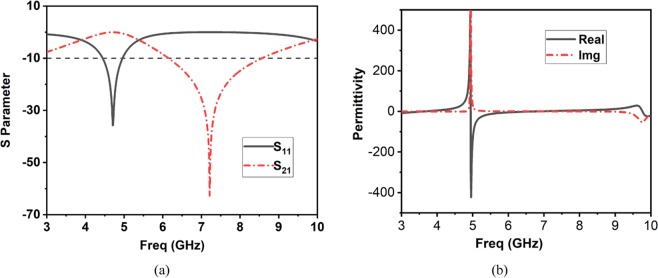
(**a**) S-parameters, (**b**) Permittivity.

**Figure 5:**
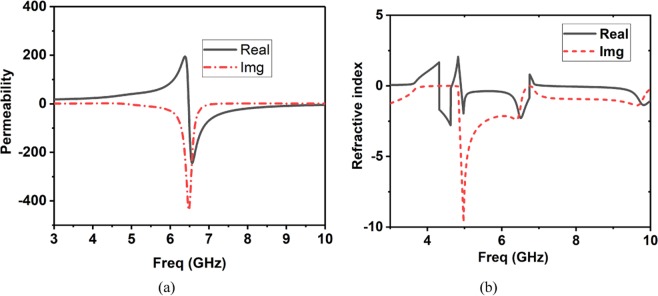
(**a**) Permeability, (**b**) Refractive Index.

The S-parameters of the unit cell are illustrated in Fig. 4a. The transmission peak is obtained at 7.2 GHz. The Nicolson-Ross-Weir method is used to extract the constitutive parameters from S-parameters. Among the parameters, permittivity (*εr*), permeability (*µ*r), and the refractive index (*ηr*) is shown in Figs. 4b, 5a,b, respectively. The equations used to calculate the values are provided in literature^52^. It is proving that the zero-index point is recorded throughout the UWB band, and the area of |re(n)| < 1 is in a wideband frequency span at the diagonal incident. It is prominent that the metasurfaces exhibit 0 < μ < 1 (MNZ) in the non-resonant regions, which can be non-dispersive through the wideband frequency. It helps the equivalent efficient refractive index to become near-zero as much of the operating band as possible. Therefore, the proposed metasurface can be represented as an index near zero metasurfaces (INZ).

#### Integration of MS with antenna

The directional radiation and high gain antenna that operates in lower frequency are preferable for microwave breast imaging system. The design starts with a conventional Vivaldi antenna. Rogers RT5880 substrate is used as the middle layer between the radiating elements in which permittivity, loss tangent, and thickness are 2.2, 0.0009 and 1.57 mm, respectively. A total of 12 MS unit is placed at the bottom radiator on an alignment of 5–3–3–1 from the top. The tapered slot is printed on the top, which acts as a radiator. To match the impedance, several side slots are etched from both ends of the bottom radiator where they help prevent reflected power. Zohu *et. al*.^53^ and Sun *et. al*.^54^ describe how metasurface can be used to enhance the gain of the directional antenna.

**Figure 6:**
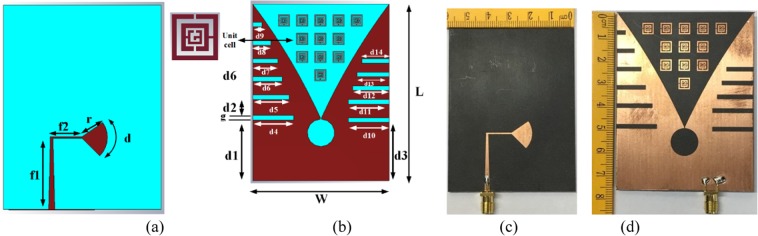
Antenna design geometry and fabricated prototype. (**a**) Top view, (**b**) Back view, (**c**) Fabricated top side and (**d**) Fabricated back side. (*Optimized parameters: f1* = *27.07 mm, f2* = *12.13 mm, r* = *9.46 mm, d* = *12.76 mm, L* = *77.72 mm, W* = *60 mm, d1* = *26.72 mm, g* = *2 mm, d2* = *7 mm, d3* = *25.72 mm, d4* = *18 mm, d5, d12* = *16 mm, d6* = *13 mm, d7* = *11 mm, d8* = *8 mm, d9* = *4 mm, d10, d11* = *18 mm, d13* = *14 mm, d14* = *12 mm*).

**Figure 7:**
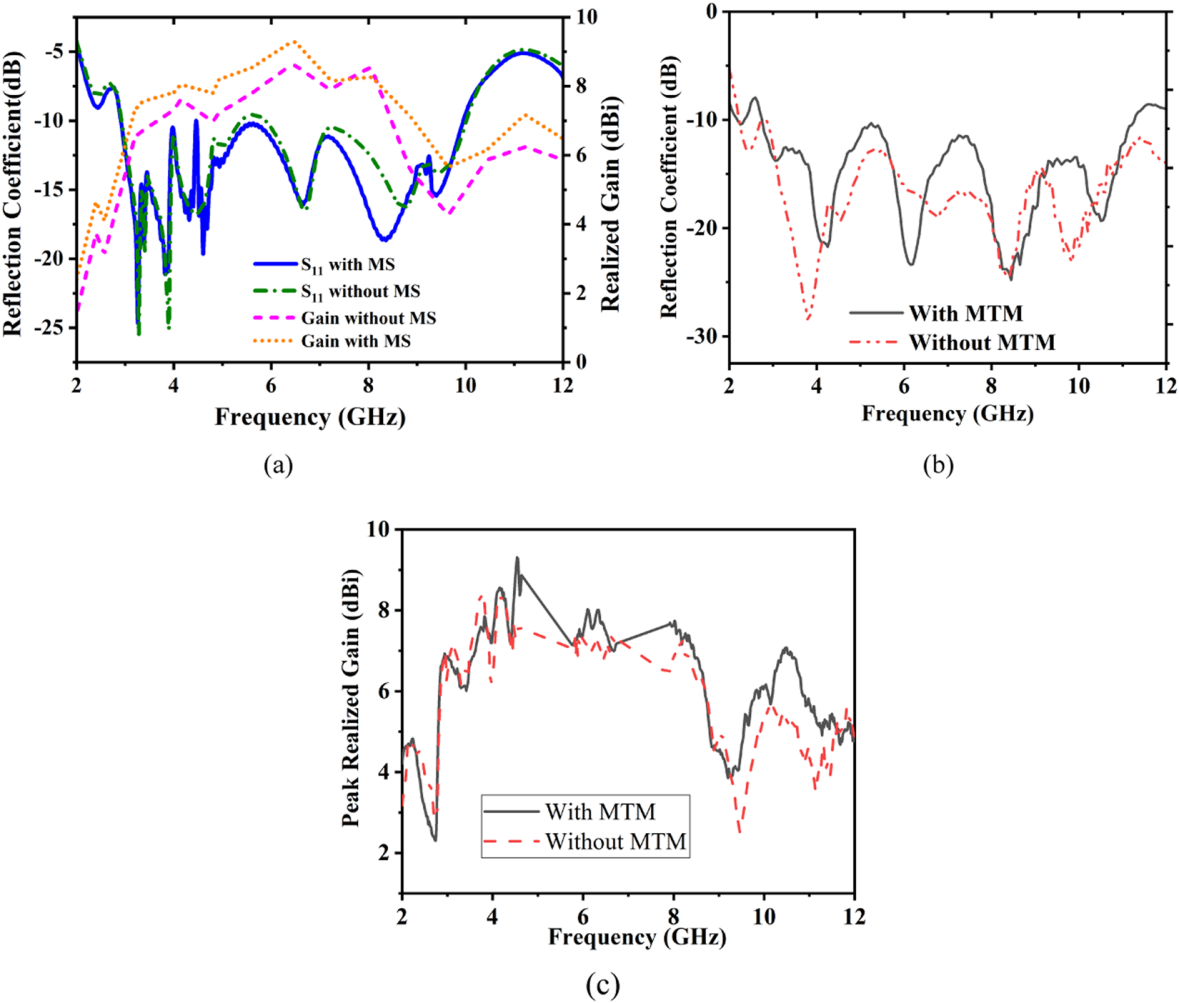
Effects of MS and without MS on reflection coefficient and realized gain (**a**) Simulated result (**b**) Measured reflection coefficient, (**c**) Measured peak realized gain.

Thus, the first target of designing high gain Vivaldi antenna based on MS is to choose proper MS structure. Figure 6 shows the design geometry and fabricated prototype with a top and bottom view. Figure 7(a) shows the effect of introducing the MS structure into the antenna. It is observed that without affecting the bandwidth, the gain has increased at a significant rate. Being an INZ structure, most of the radiated power can be transmitted through the MS that increases the electrical length of the signal and largely impacts on gain enhancements. From the measured reflection coefficient (S_11_) illustrated in Fig. 7(b), the operating bandwidth is recorded from 2.7–11.2 GHz at −10dB scale. The realized gain curve is also presented in Fig. 7(c) for both the prototype with and without MS. It is observed that the average realized gain is 7.4dBi with a maximum of 9.3dBi at 5 GHz, where the average gain was 6.9dBi with a maximum of 8.4dBi before the implementation of the metasurface at the frequency range of 2.7 GHz to 8 GHz.

**Figure 8:**
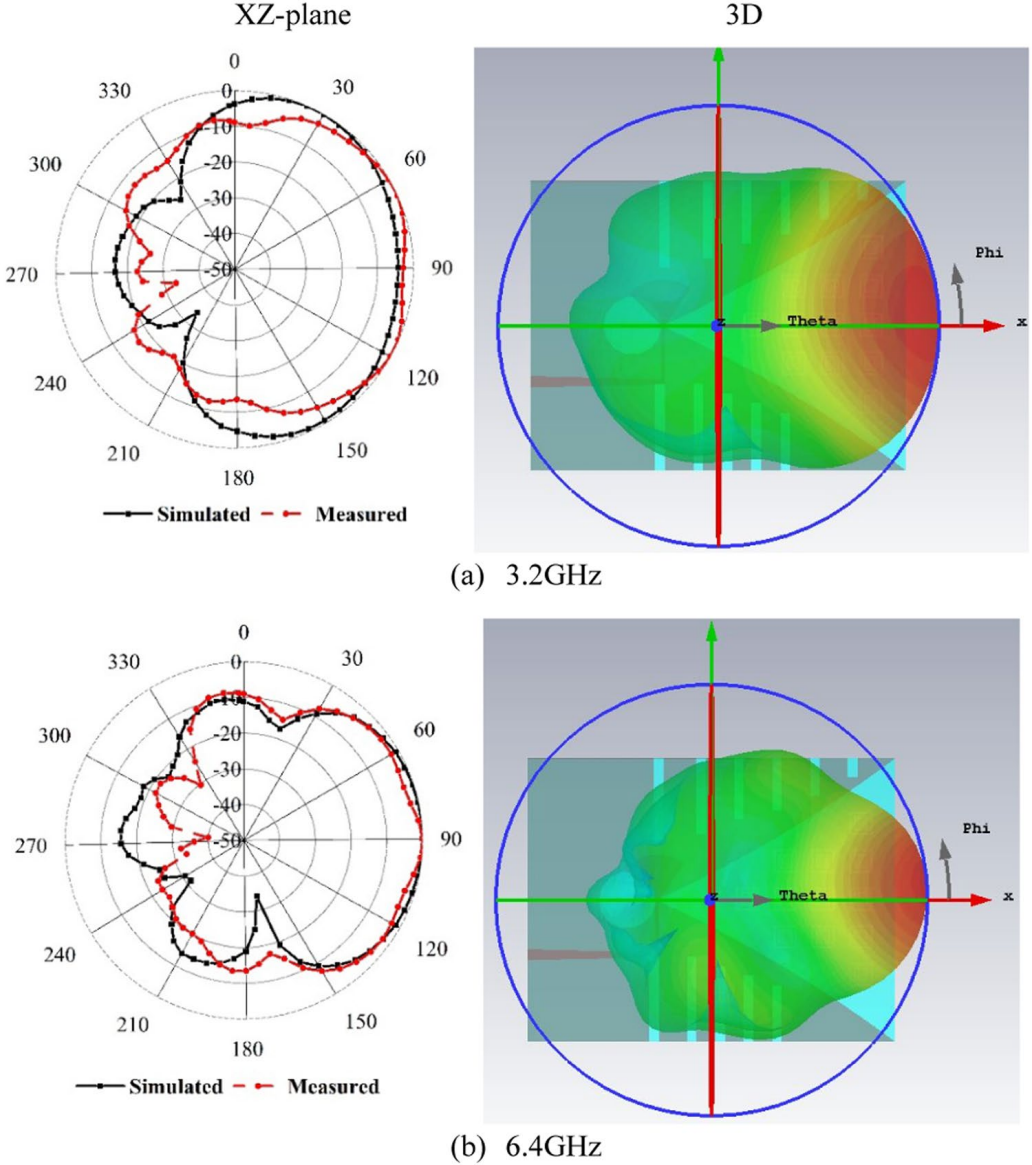
Simulated and measured radiation pattern at (**a**) 3.2 GHz and (**b**) 6.4 GHz.

To clarify the antenna radiation performance, the normalized 2D and 3D radiation pattern of the prototype is plotted in Fig. 8 for two non-identical frequencies of 3.2 and 6.4 GHz. Here, the XZ (ϕ = 0^◦^) is considered E-plane. The near field measurement shows that the antenna radiation is directional, and the primary radiation is directed towards the boresight. The main lobes are stable to the end-fire direction for both lower and higher frequencies, which is desirable for MI applications.

#### Phantom fabrication and measurement

**Figure 9:**
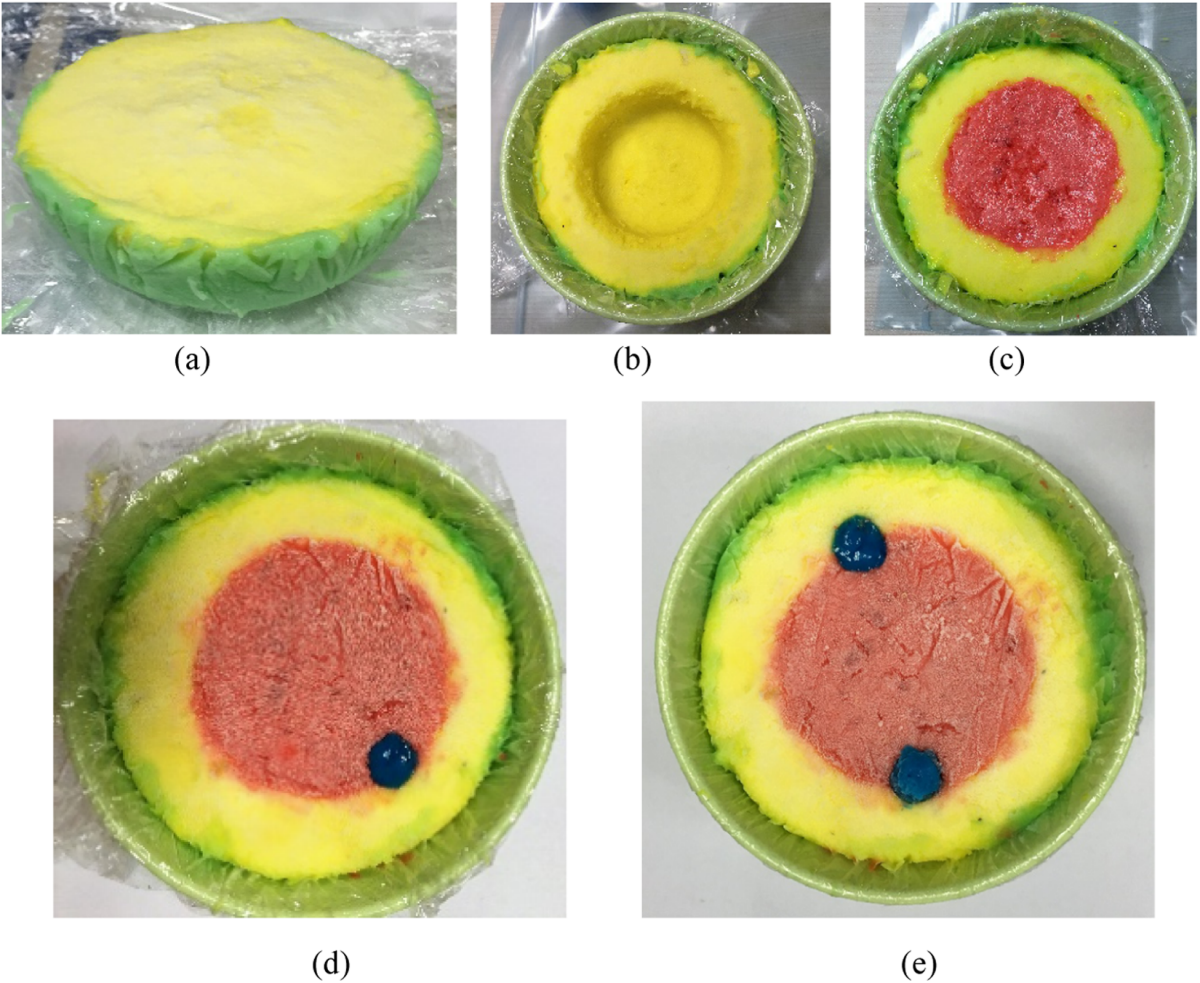
Heterogeneous phantom: (**a**) initial phantom with skin and fat layer (**b**) insertion of glandular layer (**c**) phantom with skin, fat and gland layer (Phantom A) and (**d**) phantom with single tumor inserted (Phantom B), (**e**) phantom with two tumors inserted (Phantom C).

**Figure 10:**
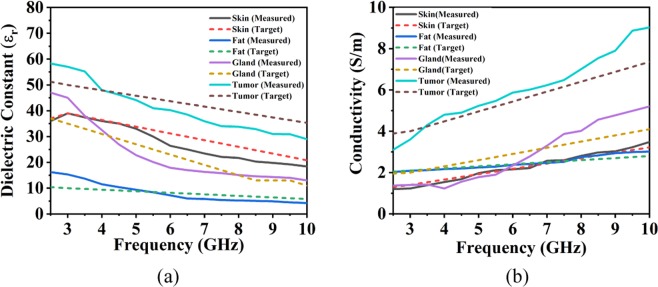
(**a**) Dielectric constant and (**b**) conductivity of each layer of the heterogeneous phantom.

The heterogeneous phantom is fabricated and measured using the methods described in literature^55^. The dielectric properties of several tissues are characterized by permittivity, which is the mean of complex-valued dielectric,

4 $$\varepsilon (\varepsilon ={\varepsilon }_{r}+i\sigma /\omega {\varepsilon }_{0})$$

where *εr* stands for dielectric constant, and *σ* means the conductivity of the tissue in contradiction of frequency. *ε*_0_ is the dielectric permittivity of vacuum, and *ω* is angular frequency. The heterogeneous phantom is fabricated. The preparation method and materials are the same, but the concentration of the materials is varied to match the dielectric constant of the tissue type being represented. For heterogeneous phantom, propylene glycol, gelatin, distilled water, safflower oil, surfactant (xanthan gum), and formalin (37% formaldehyde solution) are used. Distilled water is used to control the dielectric properties of each layer. After the preparation of each layer, the construction procedure is given in step by step format in Fig. 9. The measured dielectric constant and conductivity of each layer against frequency are shown in Fig. 10.

### Image reconstruction algorithm

The process of eliminating the reflections from the skin is critical as the scattered signal from the air-skin interface is orders of magnitude stronger in terms of power than the scattered reflections from the tumors. Rotation subtraction depends on a contrast within an original illumination, and at best single rotated illumination^56^. In such systems, the antenna array is positioned around the region of interest. The offset data is collected by rotating the phantom once the data is recorded for the original radiance.

The *S (f, tx, rx, φ)* is divided into two different matrices on the origin of *φ* being even and odd, or *S*_*even*_ *(f, tx, rx, φ*_*even*_) and *S*_*odd*_ *(f, tx, rx, φ*_*odd*_), respectively, where *φ*_*even*_ = 2,4,6, … N_*φ*_ and *φ*_*odd*_ = 1,3,5, … N_*φ*_−1. Thus, *S*_*odd*_ can be considered original illumination while *S*_*even*_ is the ‘offset’ illumination. Then, rotation subtraction is applied by just calculating the variance between the matrices.

5 $${{S}}_{{skin}{\_}{removed}}(f,\,tx,rx,\,{\phi }_{odd})={{S}}_{{odd}}(f,\,tx,rx,\,{\phi }_{odd})-{{S}}_{{even}}(f,\,tx,rx,\,{\phi }_{even}).$$

After adjusting for the skin reflections, the signals are converted to the time domain using the Inverse Fourier Transform to create *Γ(t, tx, rx, φ*_*odd*_). Then, the data in the *Γ(t, tx, rx, φ*_*odd*_) is processed via the Delay-Multiply and-Sum (DMAS) algorithm^57^ for the reconstruction of image^58^.

Typically, DAS based methods perform poorly in environments where reflections off more than one scattering object must be considered. Typically DMAS produces higher contrast images using the correlation process compared to other DAS based methods^59^. But the previous studies show that the algorithm performance is degraded in high noise environments and multi-cyst scenarios. It also has comparatively low-resolution aspects against minimum variance^60^. So, in this work, we modify the conventional DMAS algorithm by approaching delay calculation correction and iteration of the corrected values up to convergence.

The 3D Cartesian coordinates of every single point of imaging domain are demonstrated as *i’* by three matrices *C*, where the total number of points is denoted by *i’*. The patterns *A*_*Tx*_ and *A*_*Rx*_ suppress the coordinates of the transmitting and receiving antennas, individually. Being static, in the imaging domain, the rotating antennas alter their position from the points that need to be recreated. Therefore, *A*_*Txφodd*_ and *A*_*Rxφodd*_ are created by defining the antenna positions for all units while setting in initial orientation. From *C*, *A*_*Txφodd*_, and *A*_*Rxφodd*_, the *P*_*Txφodd-C*_ and *P*_*C-Rxφodd*_ are evaluated by considering the distance from every single point towards the transmitting as well as receiving antennas. In this study, air (theoretically speed of light) is considered as the background medium and divided by the overall distance to generate the proper delay, *τ(i, tx, rx, φ*_*odd*_).

6 $$\tau (i,tx,rx,{\phi }_{odd})=\frac{\sqrt{{\varepsilon }_{b}}({P}_{Tx{\phi }_{odd}-C}(i,tx,l)+{p}_{C-Rx{\phi }_{odd}}(i,rx,l))}{c}$$

where *ε*_*b*_ is the background medium dielectric constant. The delay is resulting from the projected smallest distance and reflected the signal from *C(i)*. After that, the delays are employed to the signals to deliver the appropriately delayed signals. Then, the delayed signals are pairwise multiplied, as illustrated in eq. Seven and summed to determine the scattering intensity at the given point in the region of interest. The pairwise multiplication rewards coherent signals with higher values, thus improving its performance over conventional DAS^31^.

7 $${\varUpsilon }_{DMAS}(i)={\int }_{-\infty }^{\infty }\mathop{\sum }\limits_{{\phi }_{odd}=1}^{N/2}\mathop{\sum }\limits_{tx=1}^{Tx}\mathop{\sum }\limits_{rx=1}^{Rx}\mathop{\sum }\limits_{{\phi ^{\prime} }_{odd}={\phi }_{odd}}^{N/2}\mathop{\sum }\limits_{tx^{\prime} =tx}^{Tx}\mathop{\sum }\limits_{rx^{\prime} =rx+1}^{Rx}[\begin{array}{c}\varGamma (t-\frac{\tau (i,tx,rx,{\phi }_{odd})}{\Delta t},tx,rx,{\phi }_{odd})\\ \times \varGamma (t-\frac{\tau (i,tx^{\prime} ,rx^{\prime} ,{\phi ^{\prime} }_{odd})}{\Delta t},tx^{\prime} ,rx^{\prime} ,{\phi ^{\prime} }_{odd})\end{array}]dt$$

### Calculation correction

As the dielectric materials lessen the signal propagation speed, the resulting time delay must be higher. As a result, in a region of C, a higher value of *ϒ* can be referred to as the region with increased dielectric constant. The additional time can be adjusted by suitably increasing the distances in *τ* calculations. An iterative approach is introduced for determining the most fitted delay and scattering intensity map (SIM) valuation since the adjustments of τ render to an enhanced estimation of the SIM. Though, the direct use of *ϒ* can lead the iterative process to become vulnerable and sensitive to noise levels. To overcome this scenario, a distance inverse weighted integral averaging is considered for the reproduction of leveled SIM, *ϒ* ′*(i)*. This technique is used to reflect the 3D Green function for electromagnetic signals.

8 $${\Upsilon }^{\prime} (i)={\int }_{C}\frac{{{\Upsilon }}_{DMAS}^{n-1}(i)}{1+{p}_{C-C}(i,j)}dj.$$

Then, the modified delay is calculated using the subsequent equation:

9 $$\tau ^{\prime} (i,tx,rx,{\phi }_{odd})=\tau (i,tx,rx,{\phi }_{odd})+\frac{{\Upsilon }^{\prime} (i)}{c}$$

10 $${\varUpsilon }_{DMAS}(i)={\int }_{-\infty }^{\infty }\mathop{\sum }\limits_{{\phi }_{odd}=1}^{N/2}\mathop{\sum }\limits_{tx=1}^{Tx}\mathop{\sum }\limits_{rx=1}^{Rx}\mathop{\sum }\limits_{{\phi ^{\prime} }_{odd}={\phi }_{odd}}^{N/2}\mathop{\sum }\limits_{tx^{\prime} =tx}^{Tx}\mathop{\sum }\limits_{rx^{\prime} =rx+1}^{Rx}[\begin{array}{c}\varGamma (t-\frac{\tau ^{\prime} (i,tx,rx,{\phi }_{odd})}{\varDelta t},tx,rx,{\phi }_{odd})\\ \times \varGamma (t-\frac{\tau ^{\prime} (i,tx^{\prime} ,rx^{\prime} ,{\phi ^{\prime} }_{odd})}{\varDelta t},tx^{\prime} ,rx^{\prime} ,{\phi ^{\prime} }_{odd})\end{array}]dt.$$

The SIM is reconstructed based on the new set of delays. Finally, the termination criterion inspects for convergence. Equations 9,10 are iteratively evaluated for n = 1, 2…. 7.

11 $${E}_{\varUpsilon }={\varSigma }_{\forall i}|{{\Upsilon }}_{DMAS}^{n}-{{\Upsilon }}_{DMAS}^{n-1}.$$

The iterative process is precipitately terminated when E_*ϒ*_ reduces to the anticipated level of precision as convergence has already been achieved. In this work, E_*ϒ*_ < 10^−5^ is used.

### Imaging results and numerical analysis

**Figure 11:**
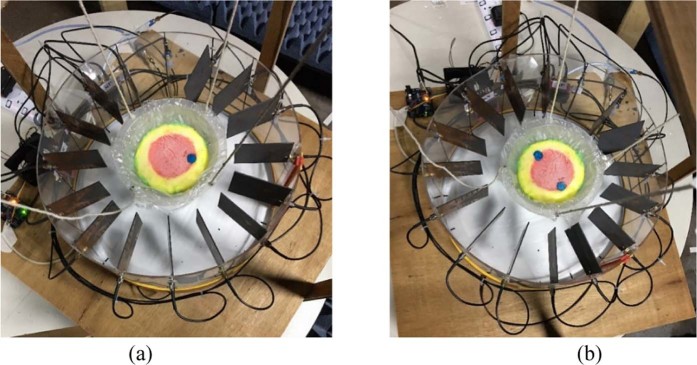
Imaging system setup for data collection.

**Figure 12:**
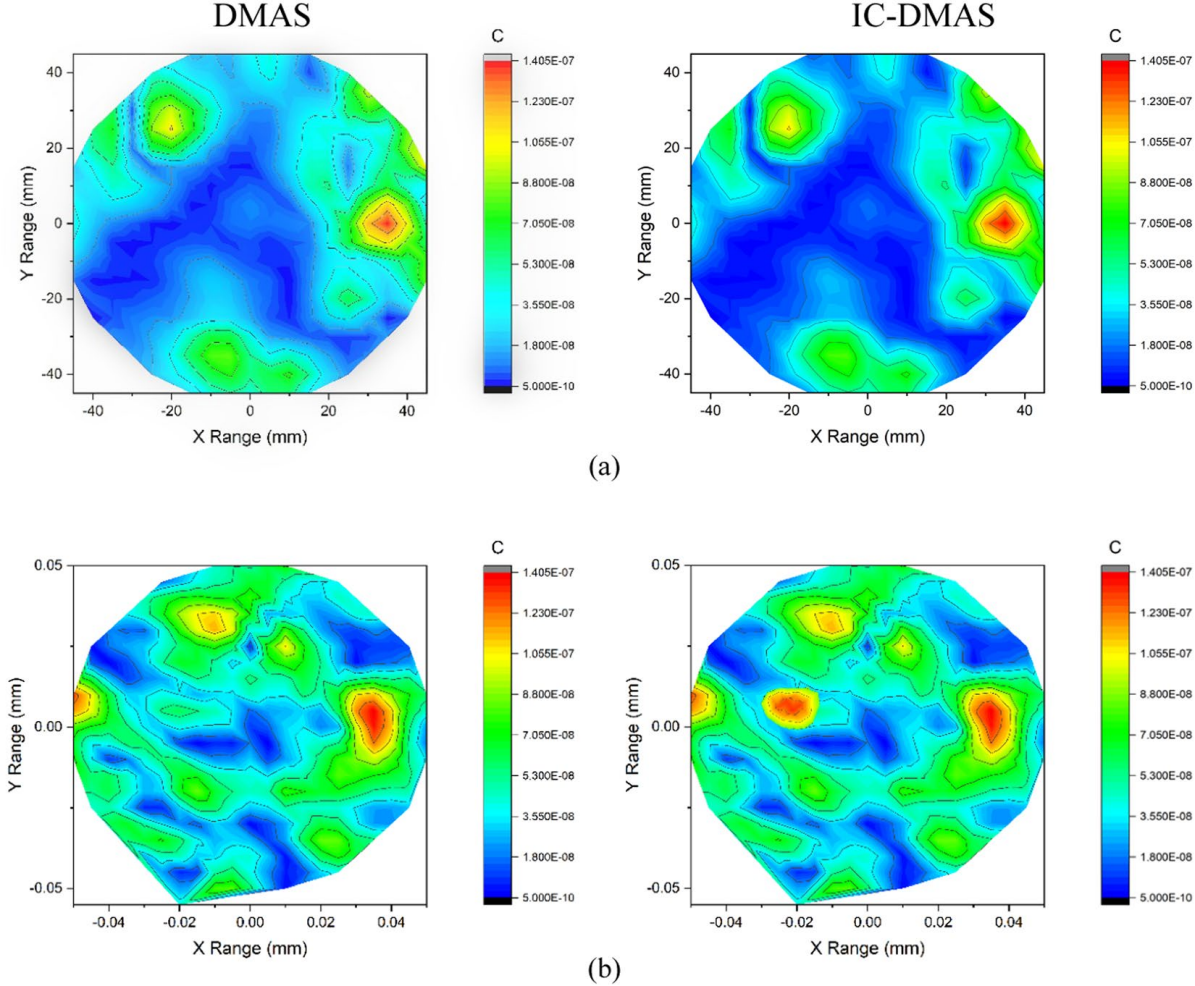
Imaging results for DMAS(Left) and Proposed IC-DMAS algorithm for two different phantoms using antenna without MS, (**a**) Phantom with a single tumor and (**b**) Phantom having two tumors.

The experimental imaging system, while taking data, is shown in Fig. 11. Two heterogeneous phantoms having different numbers of tumor inserted are considered for measurements. Sixteen antenna array elements are placed surrounding the phantom. The data is collected for further post-processing to generate images. The post-processed images are presented in Fig. 12. The tumor regions are indicated in the figure using red circles. The images on the left side show results from conventional DMAS and the images on the right show the iterative variant of DMAS results.

The iterative correction technique converged after only seven iterations as the original values of *ϒ* were low, resulting in only minor delay variation. Thus, the iterated images were enhanced slightly over DMAS on the left and the modified delay DMAS on the right. Here we present the imaging data using our two antenna prototype with and without MS. Figure 12 shows the imaging data for the antenna without MS. In Fig. 12(a), a point of high contrast to the fat material together with some lower intensity clutter for DMAS on the left. The highest contrast is formed slightly further from the center than expected resulting in some localization error. This occurs potentially due to the underestimation of the average dielectric constant of the imaging domain. The image on the right visibly shows the noise reduction attained by applying the iterative technique while enhancing the tumor response. Furthermore, the peak response is shifted more towards the center, which reduces the localization error. Figure 12(b) shows two distinct clutters indicating the presence of two tumors along with some ‘halo effect’ and ‘ghosting’ around tumors potentially due to multiple reflections from the tumors for normal DMAS. For normal DMAS the screening is unable to detect the 2^nd^ tumor. The ghosting complicates diagnosis and tumor localization significantly. The enhanced image is much clearer than the left side in terms of ghosting and also manages to detect the second tumor.

**Figure 13:**
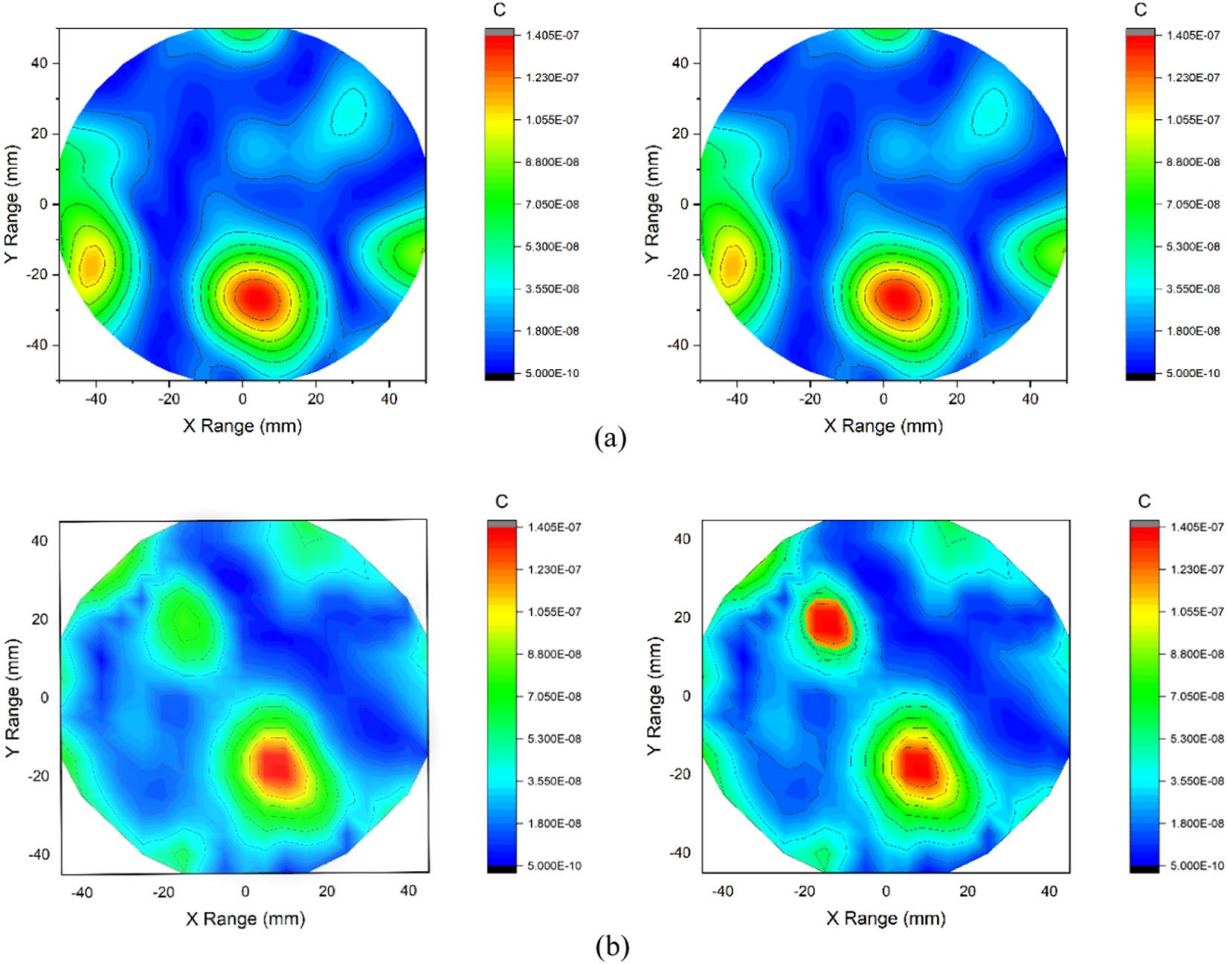
Imaging results for DMAS(Left) and Proposed IC-DMAS algorithm for two different phantoms using the proposed antenna with MS, (**a**) Phantom with a single tumor and (**b**) Phantom having two tumors.

In the 2^nd^ scenario of screening using the proposed antenna with MS the imaging results are presented in Fig. 13. More prominent and noise free images are achieved in this case. Figure 13(a) shows the single tumor response for both DMAS and IC-DMAS algorithm. The images are identical but in the 2 tumor phantom images IC-DMAS perform well by eliminating noise and ghosting and successfully detecting two tumors after removing localization errors. Furthermore, tumor size and shape are more easily recognizable. The overall signal to mean ratio (SMR) results show noticeable improvements for tumor detection in both cases (with and without MS antenna) using the iterative method, as highlighted in Table 1 and Table 2.

**Table 1:** Signal to Mean Ratio, in dB, for phantoms B, C for antenna without MS.

Algorithm	DMAS	IC-DMAS
Phantom B	5.2694	11.7459
Phantom C	12.6783	25.8654

**Table 2:** Signal to Mean Ratio, in dB, for phantoms B, C for antenna with MS.

Algorithm	DMAS	IC-DMAS
Phantom B	7.9450	16.4342
Phantom C	14.8536	33.9022

The performance of the reconstruction algorithms can be evaluated by the Signal to Mean ratio (SMR). The calculation is shown in the following equation.

12 $$SMR=\frac{MaximumTumorEnergy}{MeanEnergy}$$

The SMR defines the ratio of maximum backscattered tumor energy to the average energy response from backscattering signals at the same sample.

The signal to mean ratio for the DMAS and IC-DMAS is compared to the modified algorithm in Table 1 and Table 2 below. The signal to mean ratio is substantially improved in both phantoms studied. The signal to mean ratio is calculated by averaging the values of *ϒ* across the volume of the tumor and determining its ratio to the overall average of *ϒ* in the reconstructed image. The antenna with MS shows better performance in this numerical study for both single and double tumor detection scenarios.

A detailed comparison of the proposed system with literature is presented in Table 3. The parameters considered of comparison are the type of antenna, operating frequency, No. of antenna element/scanning position, type of system, Frequency/Time-domain measurement, and Types of phantom and number of tumors detected. The proposed system can successfully detect multiple objects.

**Table 3:** Comparison of the different imaging system with the proposed one.

Investigator	Antenna Type	Freq. (GHz)	Elements/Position	Fixed/Movable	Frequency/Time domain	Imaging Method	Phantom and tumor object
^14^	Slotted patch	3.5–15	4 × 4 Single element	Fixed Switching Matrix	Frequency domain	Confocal Imaging	Simulated phantom Yes
^16^	UWB transceiver	3–10	16 Element arrays	Fixed Switching Matrix	Time-domain	DAS Simulation only	No No No
^19^	CPW feed Monopole	2–4	16 Elements array 16 × 15 scanned position	Fixed Switching Matrix	Time-domain	DMAS	Yes Yes Single
^20^	Slotted antipodal Vivaldi antenna	3.01–11	Two elements 2 × 50 position	Fixed Platform rotated	Time-domain	DMAS	Yes Yes Single
^21^	UWB Flexible antenna	4–6	2 Element arrays	Fixed	Frequency domain	DMAS	Commercial Single
^22^	UWB antenna	3.1–10.6	16 Element arrays	Movable	Time domain	Confocal Imaging	Patient single
^29^	Balanced antipodal Vivaldi antenna	1–13	36 Single element scanned position	Fixed Tank Rotate monostatic	Frequency domain	TSAR (Tissue Sensing Adaptive Radar)	Sample Tissue No
^31^	Tapered and transmission loaded antenna	2–8	16 element Array 16 × 15 scanned position	Fixed Switching Matrix	Time-domain	DMAS (Delay Multiply and Sum Algorithm)	Lab-based breast phantom Yes Single
^36^	CPW feed EBG structure Antenna	3.1–7.6	2 antenna elements 2 × 120 scanned position	Fixed Platform rotated	Frequency Domain	DMAS	Commercial phantoms Single tumor object
^61^	Antipodal Vivaldi	2.5–11	9 antenna arrays 8 × 50 position	Movable	Frequency Domain	IC-CF-DAS	Lab made Multiple object
Proposed	Index Near-Zero Metasurface Loaded High Gain Antenna	2.7–8.0	16 antenna arrays 64 × 50 scanned position	Movable multistatic	Time and Frequency Domain	IC-DMAS	Lab-made realistic heterogeneous phantom 1, 2 tumor objects

## Conclusion

The design of a microwave imaging system comprising of metasurface loaded high gain antenna array is presented in this work. The proposed antenna achieved high gain with directive radiation after loading 12 complementary split-ring resonators (CSRR) structure index-near-zero metasurface on the ground plane. The antenna operates between 2.7–11.2 GHz, which covers the operating band of the switching matrix (10 MHz–8 GHz), and maximum gain 9.2dBi is achieved at 5 GHz. A cylindrical arrangement of 16 antenna elements fitted on a mechanical rotating platform is used for screening. A set of realistic heterogeneous breast phantoms is fabricated, measured and compared with the real human breast tissue dielectric properties. After achieving a good agreement, the phantoms are used for testing the system efficiency. A single PC unit controls the rotating and switching mechanism through a customized MATLAB based imaging program. An iterative variant of delay multiply and sum (IC-DMAS) algorithm is used for getting a clearer image and remove skin artifact, ghosting and halo effect around the tumors. After collecting the scattered signal from the proposed system, the data is further processed and analyzed. The imaging results from the testing system are presented and the MS based system can detect multiple abnormalities. By using the proposed INZ metasurface loaded antenna, 16 array element system, and IC-DMAS algorithm, the work presented in this article can detect tumor clusters inside the human breast that can be efficient, viable and low-cost complement to the conventional imaging systems.
